# Association between ambient air pollution and outpatient visits of cardiovascular diseases in Zibo, China: a time series analysis

**DOI:** 10.3389/fpubh.2024.1492056

**Published:** 2025-01-08

**Authors:** Yamei Wang, Shaoning Qu, Ting Li, Liang Chen, Liping Yang

**Affiliations:** ^1^Department of Epidemiology, School of Public Health, Cheeloo College of Medicine, Shandong University, Jinan, China; ^2^Department of Emergency, Qilu Hospital of Shandong University, Jinan, China

**Keywords:** ambient air pollutants, coronary heart disease, stroke, arrhythmia, Zibo

## Abstract

**Introduction:**

Facing Mount Tai in the south and the Yellow River in the north, Zibo District is an important petrochemical base in China. The effect of air pollution on cardiovascular diseases (CVDs) in Zibo was unclear.

**Methods:**

Daily outpatient visits of common CVDs including coronary heart disease (CHD), stroke, and arrhythmia were obtained from 2019 to 2022 in Zibo. Air pollutants contained fine particulate matter (PM_2.5_), inhalable particulate matter (PM_10_), nitrogen dioxide (NO_2_), sulfur dioxide (SO_2_), ozone (O_3_), and carbon monoxide (CO). Distributed lag non-linear models (DLNM) including single-pollutant model in single-day (lag0-lag7) and cumulative-days (lag01-lag07), concentration-response curve, subgroup analysis, and double-pollutant model were utilized to examine the relationships of daily air pollutants on CHD, stroke, and arrhythmia. Meteorological factors were incorporated to control confounding.

**Results:**

In single-pollutant model, NO_2_ was positively associated with CHD, stroke and arrhythmia, with the strongest excess risks (ERs) of 4.97% (lag07), 4.71% (lag07) and 2.16% (lag02), respectively. The highest ERs of PM_2.5_ on CHD, stroke and arrhythmia were 0.85% (lag01), 0.59% (lag0) and 0.84% (lag01), and for PM_10_, the ERs were 0.37% (lag01), 0.35% (lag0) and 0.39% (lag01). SO_2_ on CHD was 0.92% (lag6), O_3_ on stroke was 0.16% (lag6), and CO on CHD, stroke, and arrhythmia were 8.77% (lag07), 5.38% (lag01), 4.30% (lag0). No threshold was found between air pollutants and CVDs. The effects of ambient pollutants on CVDs (NO_2_&CVDs, PM_2.5_&stroke, PM_10_&stroke, CO&stroke, CO&arrhythmia) were greater in cold season than warm season. In double-pollutant model, NO_2_ was positively associated with CHD and stroke, and CO was also positively related with CHD.

**Conclusion:**

Ambient pollutants, especially NO_2_ and CO were associated with CVDs in Zibo, China. And there were strong relationships between NO_2_, PM_2.5_, PM_10_, CO and CVDs in cold season.

## 1 Introduction

The incidence and mortality of cardiovascular diseases (CVDs) are continuously rising, as revealed by the China Cardiovascular Disease Report 2022. There are approximately 330 million CVD patients, including 13 million cases of stroke, 11.4 million cases of coronary heart disease (CHD), and 4.87 million cases of atrial fibrillation in China. CVDs account for over 40% of the total deaths, making it the leading cause of death among the population (1). A staggering 6.67 million deaths worldwide, accounting for 12% of total deaths, were attributed to air pollution. Ambient air pollutants rank as the fourth leading risk factor for the burden of CVDs, as reported by the Global Burden of Disease (GBD) (2). Ambient pollution has become one of the biggest threats of our time (3). Environmental air pollutants are important health hazards (4). Numerous domestic and international studies have indicated that exposure to ambient air pollutants could lead to various cardiovascular health outcomes. Short-term exposure to fine particulate matter (PM_2.5_), inhalable particulate matter (PM_10_), and nitrogen oxides (NO_x_) were associated with increased risks of myocardial infarction and stroke (5). Sulfur dioxide (SO_2_) and nitrogen dioxide (NO_2_) significantly increased the daily number of cardiovascular hospitalizations in areas with a low level of air pollution (6). Various studies have shown that exposure to carbon monoxide (CO) was associated with mortality, hospital admissions, and outpatient visits of CVDs (7–12). However, there are discrepancies in the natural environment and population structure across different regions.

Zibo, a modern industrial area and an important petrochemical base, is one of the “2 + 26” cities comprised by of Beijing City, Tianjin City, and 26 surrounding cities-the area with the worst air quality in China. In December 2021, Zibo municipal government declared that various industrial furnaces and kilns in Zibo, accounting for more than one-fifth in Shandong Province, could emit large amounts of pollutants. However, no existing study in the region exploring the risks of ambient air pollutants on CHD, stroke and arrhythmia has been reported. Therefore, this study focused on investigating the effects of ambient air pollutants on common CVDs, and identifying key pollutants that had significant impacts on CHD, stroke and arrhythmia from 2019 to 2022 in Zibo.

## 2 Materials and methods

### 2.1 Study area

**Figure 1:**
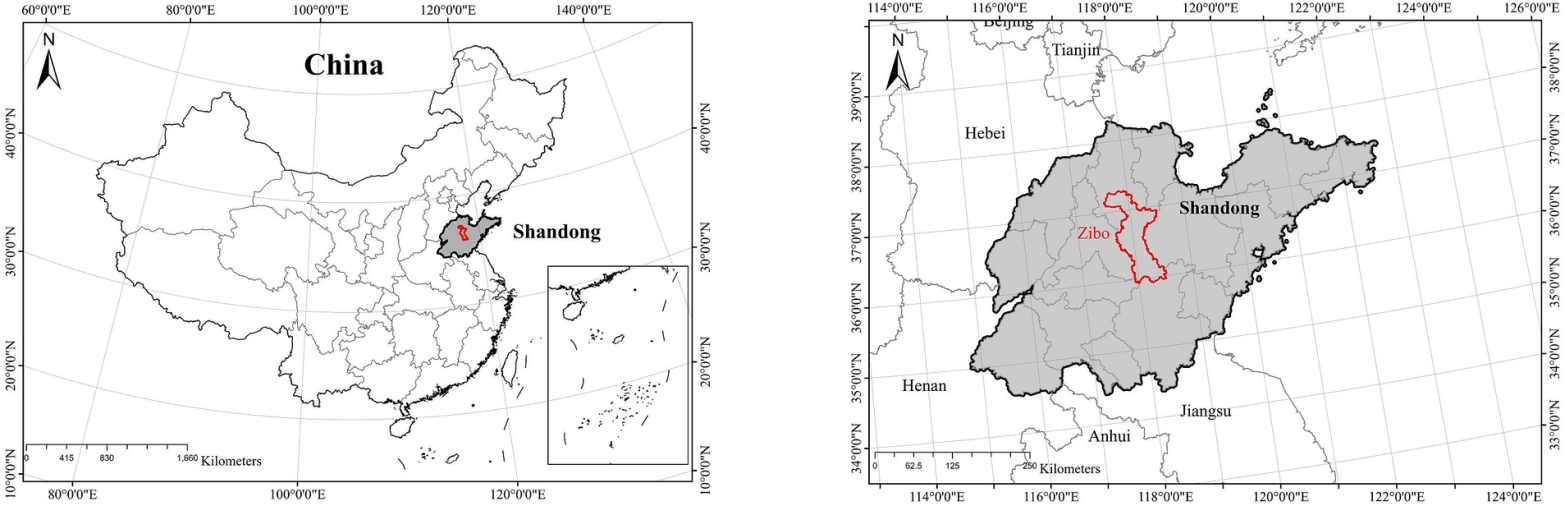
The location of Zibo District, Shandong Province, China.

Zibo district, located in the center of Shandong Province in eastern China (Figure 1), is one of the core cities of the Shandong Peninsula urban agglomeration. It is situated between 35°55′20″ and 37°17′14″ north latitude, 117°32′15″ and 118°31′00″ east longitude, bordered by Mount Tai to the south and the Yellow River to the north. The terrain of Zibo is high in the south and low in the north, with an elevation difference of over 1, 000 meters. Zibo is situated with the warm temperate zone and experiences a semi-humid and semi-arid continental climate. These topographic and climatic conditions restrict the diffusion of ambient air pollutants. The district covered a total area of 5, 965 square kilometers and had a resident population of 4.71 million people as of the end of 2022, as released by the 2023 Statistical Yearbook of Zibo.

### 2.2 Data

#### 2.2.1 Data on daily outpatient visits

Data on daily outpatient visits of CHD, stroke, and arrhythmia from January 1, 2019 to December 31, 2022 were from all hospitals with hospital information system (HIS) in Zibo. The data were extracted based on the corresponding diagnosis codes from the International Statistical Classification of Diseases and Related Health Problems 10th Revision (ICD-10), focusing on CHD (I20-I25), stroke (I60-I61and I63-I64), and arrhythmia (I44-I49). The dataset contained diagnosis codes, outpatient codes, outpatient visit dates, age, gender, and local resident or not. Inclusion criteria: diseases with ICD-10 codes including I20-I25, I44-I50, I60-I61 and I63-I64. Exclusion criteria: (1) patients with residential address outside Zibo; (2) records without complete information.

#### 2.2.2 Data on ambient air pollutants

Data on ambient air pollutants were from the China National Environmental Monitoring Centre (CNEMC), the National Urban Air Quality Real-time Publishing Platform. The data were collected from seven state-controlled monitoring sites in Zibo, which included People’s Park, Shuangshan, Meteorological Station, Putian Park, New District, Sankin Group, Dongfeng Chemical Factory. Pollutants including NO_2_, PM_2.5_, PM_10_, SO_2_, CO were reported as 24-h average concentrations, while ozone O_3_ was measured as the daily maximum 8-h concentration.

#### 2.2.3 Data on meteorology factors

Meteorological data were obtained from China Meteorological Administration, including temperature (°C), relative humidity (%), and wind speed (m/s).

### 2.3 Statistical methods

#### 2.3.1 Descriptive statistical analysis

Continuous variables, including daily outpatient visits, ambient air pollutants, and meteorological factors, were described using mean, standard deviation (SD), and percentiles (1st, 25th, 50th, 75th, and 99th). Categorical data were characterized by frequency (n) and composition ratio (%). Spearman′s correlation coefficient was used to represent the correlation among ambient pollutants and meteorological factors.

#### 2.3.2 Statistical analysis

A distributed lag non-linear model (DLNM) was utilized to examine the associations between air pollutants (NO_2_, PM_2.5_, PM_10_, SO_2_, O_3_ and CO) and outpatient visits of CVDs. The main strength of DLNM was its ability to concurrently model exposure-response and lag-response relationships between exposure and outcome, thereby elucidating the relationship between exposure and outcome across both exposure and lag dimensions (13). The basic structure of the model was outlined as follows,


$$\begin{array}{l} \log\left[E\left(Y_{t}\right)\right]=\alpha +cb\left(\mathrm{Pollutan}t_{t}\right)+cb\left(T_{t}\right)\\ +cb\left(RH_{t}\right)+cb\left(\mathrm{Win}d_{t}\right)+\mathrm{ns}\left(t_{\mathrm{time}},df=7/\mathrm{year}\right)\\ +V_{DOW}+V_{\mathrm{holiday}} \end{array}$$

where *Y_t_* represented the actual value of outpatient visits on day t, *E* (*Y_t_*) represented the expected value of outpatient visits, *α* represented the intercept, and *cb* represented the cross-basis function. *Pollutant*, *T_t_*, *RH_t_* and *Wind_t_* represented the values of ambient air pollutants, temperature, relative humidity, and wind speed on day t, respectively. Additionally, *t_time_* was incorporated in the form of a natural cubic spline function (*ns*) to control for long-term and seasonal trends, with the degree of freedom (*df*) set to 7. In the cross-basis between temperature, relative humidity, wind speed and CVDs, the *df* was set to 3. *V_DOW_* and *V_holiday_* were dummy variables to control the effects of weekday and holiday. Finally, a linear approach was applied to fit the concentration-response relationship (14, 15).

The effect of ambient air pollution on cardiovascular events generally persisted for 3 to 6 days, as shown by previous studies (16–18). To adequately examine the lag effects of ambient pollutants, the maximum lag day in the study was set to 7. Additionally, the effects of air pollutants on CVDs can manifest as both single and cumulative lag effects, therefore, single-pollutant model was used to examine the effects of ambient air pollutants in single-day and cumulative-days on CVDs. Lag0-lag7 was used to denote single-day and lag01-lag07 to denote cumulative-days. Double-pollutant model was developed by adjusting for other air pollutants, including NO_2_, PM_2.5_, PM_10_, SO_2_, O_3_ and CO.

#### 2.3.3 Subgroup analysis and sensitivity analysis

To gain further understanding of the associations between air pollutants and outpatient visits of CVDs, subgroup analysis was performed based on gender, age, and season. Gender groups were conducted for males and females. Age groups were stratified into less than 60 years old (<60 years) and those aged 60 years or older (≥60 years). Seasons were categorized as warm season (April to September) and cold season (October to March). Z-tests were used to assess statistical differences between subgroups with the following formula,


$$Z=\frac{\beta_{1}-\beta_{2}}{\sqrt{SE_{1}^{2}+SE_{2}^{2}}}$$

where *β_1_* and *β_2_* were the regression coefficients of subgroups, and *SE_1_* and *SE_2_* were the corresponding standard errors (8, 14, 19).

Sensitivity analysis was conducted by adjusting the *df* of temperature, relative humidity, and wind speed in the cross-basis to 4 and 5, respectively. Additionally, the *df* of the *ns*, used to control for long-term and seasonal trends, was modified to 8.

Zero value of daily air pollutant concentration was used as a reference, relative risk (RR) and its 95% confidence interval (95%CI) were used to assess the associations between air pollutants and CVDs. To facilitate the interpretation and comparison of data, RR was converted into excess risk (ER), using the following equation: $ER=\left(RR-1\right)*100\%$ . ER (95%CI) indicated percentage changes in outpatient visits of CVDs associated with every 10 μg/m^3^ increase of ambient air pollutants (with CO represented as 1 mg/m^3^). All analyses were two-sided tests with a significance level of *α* = 0.05 and performed in R 4.1.3.

## 3 Results

### 3.1 Data description

The annual average concentrations of NO_2_, PM_2.5_, PM_10_, SO_2_, O_3_, and CO were 36.9 μg/m^3^, 51.2 μg/m^3^, 91.3 μg/m^3^, 16.9 μg/m^3^, 113.4 μg/m^3^, and 0.9 mg/m^3^, respectively (Table 1). A total of 1, 089, 136 outpatient visits were documented. The highest number of outpatient visits was CHD, totaling 809, 792 cases, which accounted for 74.3%, and others included stroke with 142, 625 cases (13.1%), and arrhythmia with 136, 719 cases (12.6%). Statistical differences were found in gender and age subgroups of CHD, stroke and arrhythmia.

**Table 1:** Summary statistics of daily outpatient visits of CHD, stroke, arrhythmia, ambient air pollutants, and meteorological factors in Zibo, China, 2019–2022.

Variable	Mean	SD	Percentiles					*p* value
			P_1_	P_25_	P_50_	P_75_	P_99_	
Daily outpatient visits								
CHD	555	212	106	399	564	717	995	
Gender								
Male	242	92	45	178	246	311	447	<0.001
Female	313	122	62	225	318	406	573	
Age (year)								
<60	220	78	43	165	224	275	402	<0.001
≥60	335	143	58	216	346	447	623	
Stroke	98	43	16	68	92	125	206	
Gender								
Male	46	22	7	30	42	58	101	<0.001
Female	53	24	7	36	50	67	109	
Age (year)								
<60	43	20	7	29	41	54	95	<0.001
≥60	56	28	7	36	50	69	132	
Arrhythmia	94	37	15	68	91	119	186	
Gender								
Male	43	18	5	29	40	53	90	<0.001
Female	52	21	7	37	50	65	102	
Age (year)								
<60	42	20	6	28	39	52	105	<0.001
≥60	52	23	8	36	50	65	117	
Ambient air pollutants								
NO_2_ (μg/m^3^)	36.9	15.3	12.0	25.0	34.0	47.0	82.0	
PM_2.5_ (μg/m^3^)	51.2	34.9	10.0	29.0	41.0	62.0	184.0	
PM_10_ (μg/m^3^)	91.3	55.9	15.7	57.0	79.0	112.0	270.6	
SO_2_ (μg/m^3^)	16.9	8.4	5.0	11.0	15.0	21.0	47.0	
O_3_ (μg/m^3^)	113.4	55.2	19.0	69.0	106.0	153.0	247.3	
CO (mg/m^3^)	0.9	0.4	0.3	0.6	0.8	1.1	2.4	
Meteorological factors								
Temperature (°C)	14.2	10.6	−6.5	5.4	14.6	23.7	31.5	
Relative humidity (%)	64.4	16.4	29.0	52.0	66.0	77.0	96.0	
Wind speed (m/s)	1.7	0.8	0.5	1.1	1.6	2.2	4.2	

**Figure 2:**
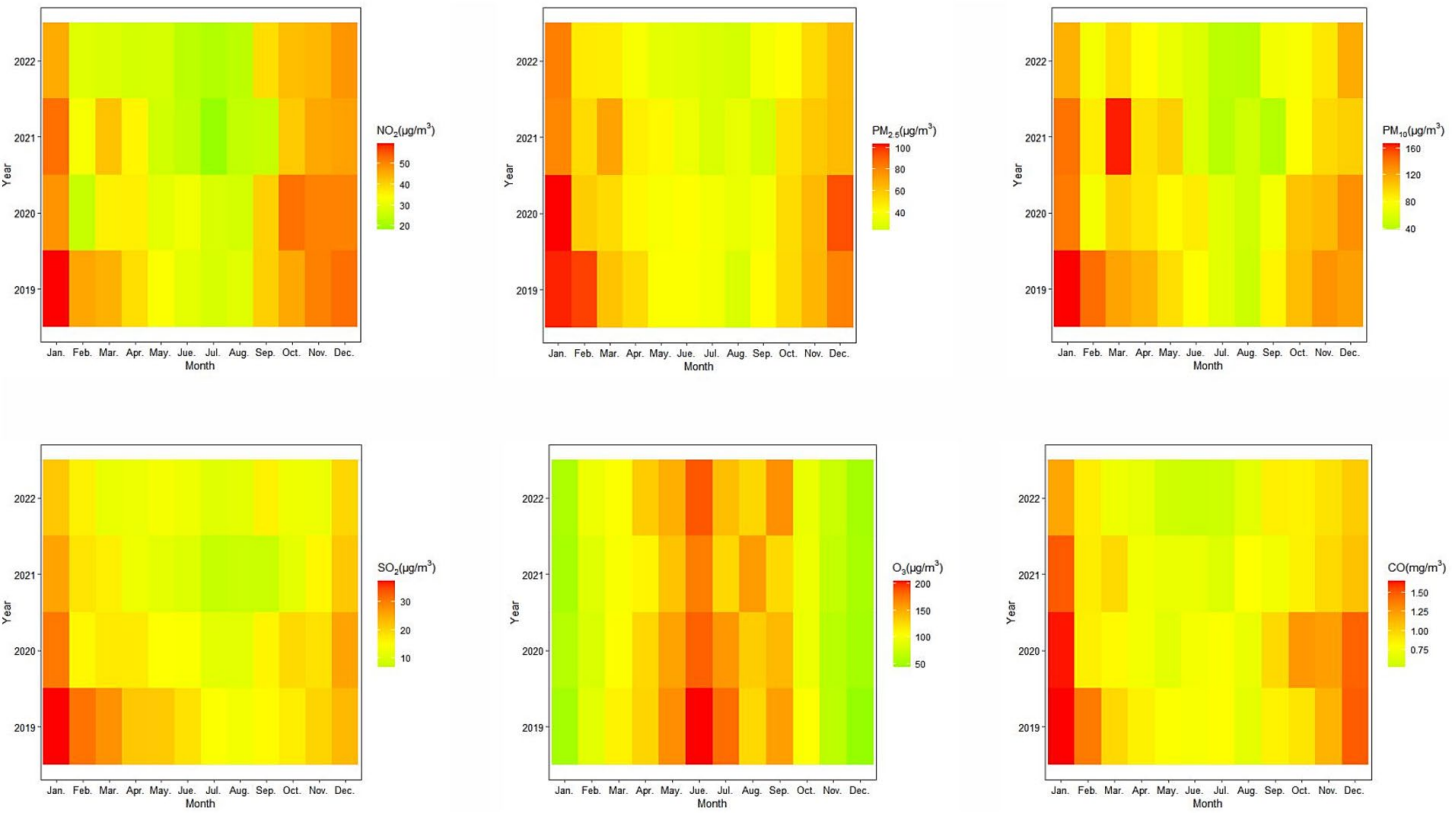
Monthly variations of ambient air pollutants (NO_2_, PM_2.5_, PM_10_, SO_2_, O_3_, and CO) in Zibo, China, 2019–2022.

There was obvious seasonality in concentrations of air pollutants, the concentrations of NO_2_, PM_2.5_, PM_10_, SO_2_, and CO were higher in winter and lower in summer, while O_3_ showed an opposite pattern (Figure 2). In addition, the concentrations of NO_2_, PM_2.5_, PM_10_, SO_2_ and CO showed a decreasing trend from 2019 to 2022, but the annual change of O_3_ was less obvious. Compared to PM_2.5_, the duration of higher NO_2_ concentration was extended, peaking from January to March and from September to December.

### 3.2 Regression results

#### 3.2.1 Spearman′s correlation analysis

NO_2_, PM_2.5_, PM_10_, SO_2_ and CO were positively correlated with each other, while these pollutants mentioned above were negatively correlated with O_3_, temperature, and wind speed. O_3_ was positively correlated with temperature and wind speed, but it was negatively correlated with relative humidity. Temperature was positively correlated with relative humidity and wind speed. Additionally, relative humidity was positively correlated with CO, but negatively associated with PM_10_, SO_2_, O_3_, wind speed (see Supplementary Table S1).

#### 3.2.2 Single-pollutant model

**Figure 3:**
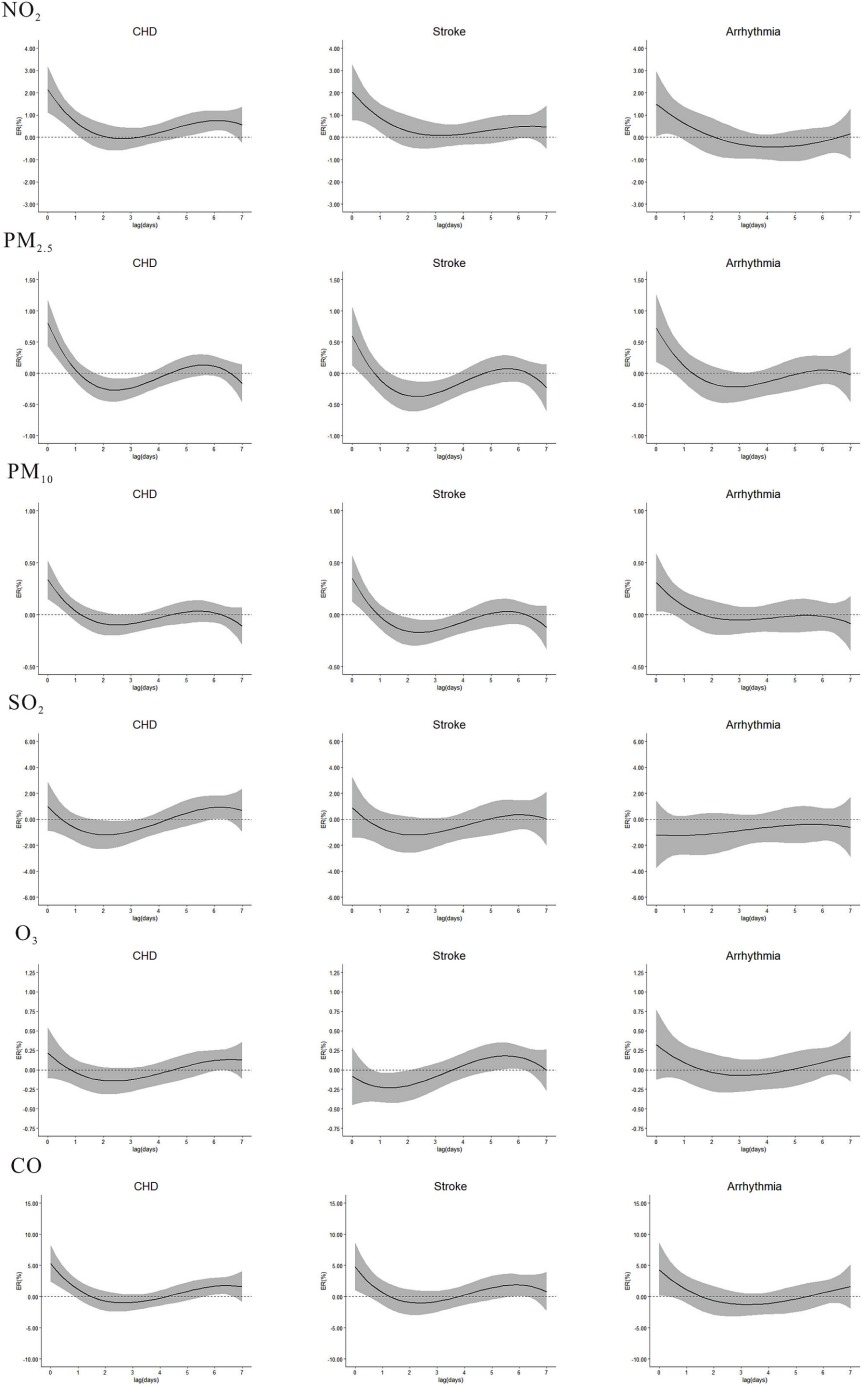
Single excess risks (ER, 95%CI) in outpatient visits of CHD, stroke, and arrhythmia associated with NO_2_, PM_2.5_, PM_10_, SO_2_, O_3_, and CO in single-pollutant model, at lag0-lag7.

The single effects of NO_2_, PM_2.5_, PM_10_ and CO on CHD, stroke and arrhythmia peaked on the current day and then gradually declined, becoming insignificant after approximately 1–2 days (Figure 3). The strongest single ERs of NO_2_ on CHD, stroke and arrhythmia were 2.14% (95%CI: 1.12, 3.18), 2.02% (95%CI: 0.78, 3.27) and 1.49% (95%CI: 0.05, 2.95) at lag0, respectively. The strongest ERs between PM_2.5_ and mentioned above diseases were 0.80% (95%CI: 0.43, 1.18), 0.59% (95%CI: 0.13, 1.06) and 0.72% (95%CI: 0.13, 1.06) at lag0, and for PM_10_, the ERs were 0.34% (95%CI: 0.15, 0.52), 0.35% (95%CI: 0.13, 0.57) and 0.31% (95%CI: 0.03, 0.59) at lag0. CO on CHD, stroke and arrhythmia were 5.26% (95%CI: 2.39, 8.22), 4.74% (95%CI: 0.99, 8.63), and 4.30% (95%CI: 0.14, 8.64) at lag0. SO_2_ was only associated with CHD, with the strongest ER of 0.92% (95%CI: 0.03, 1.81) at lag6. O_3_ was solely associated with stroke, with the highest ER of 0.16% (95%CI: 0.01, 0.31) at lag6.

**Figure 4:**
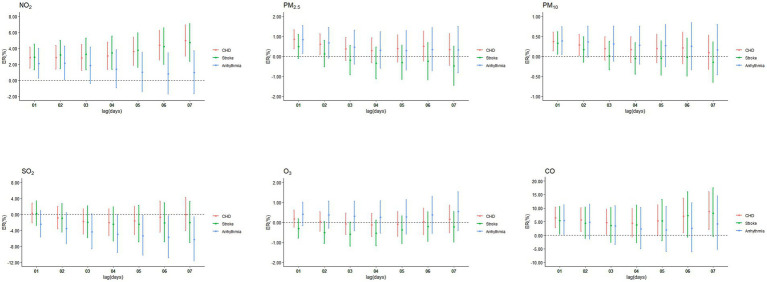
Cumulative excess risks (ER, 95%CI) in outpatient visits of CHD, stroke, and arrhythmia associated with ambient air pollutants in single-pollutant model, at lag01-lag07.

NO_2_ was significantly associated with CHD and stroke during the entire cumulative lag period (Figure 4), the highest cumulative effects of NO_2_ on CHD, stroke and arrhythmia were at lag07 (ER = 4.97, 95%CI: 3.03, 6.94), lag07 (ER = 4.71, 95%CI: 2.37, 7.10), and lag02 (ER = 2.16, 95%CI: 0.12, 4.25). The highest ERs of PM_2.5_ on CHD and arrhythmia were 0.85% (95%CI: 0.38, 1.33) and 0.84% (95%CI: 0.15, 1.53) at lag01. For PM_10_, the ERs on CHD, stroke and arrhythmia were 0.37% (95%CI: 0.14, 0.60), 0.33% (95%CI: 0.05, 0.61) and 0.39% (95%CI: 0.04, 0.74) at lag01. The ERs of CO on CHD and stroke were at lag07 (ER = 8.77, 95%CI: 2.06, 15.91) and lag01 (ER = 5.38, 95%CI: 0.45, 10.54). No cumulative effect of PM_2.5_ on stroke, CO on arrhythmia, SO_2_ and O_3_ on CHD, stroke and arrhythmia was observed. ERs of NO_2_, PM_2.5_, PM_10_, SO_2_, O_3_ and CO in single-pollutant models of single-day and cumulative-days on outpatient visits of CHD, stroke, and arrhythmia peaked at different lag days (Table 2).

**Table 2:** The largest excess risks (95%CI) and the lags of NO_2_, PM_2.5_, PM_10_, SO_2_, O_3,_ and CO on outpatient visits of CHD, stroke, and arrhythmia, in single-pollutant model.

Pollutant	Increase	CHD		Stroke		Arrhythmia	
		ER (95%CI)	Lag (day)	ER (95%CI)	Lag (day)	ER (95%CI)	Lag (day)
**NO**_**2**_	**10 μg/m** ^**3**^	**4.97 (3.03, 6.94)**	07	**4.71 (2.37, 7.10)**	07	**2.16 (0.12, 4.25)**	02
**PM**_**2.5**_	**10 μg/m** ^**3**^	**0.85 (0.38, 1.33)**	01	**0.59 (0.13, 1.06)**	0	**0.84 (0.15, 1.53)**	01
**PM**_**10**_	**10 μg/m** ^**3**^	**0.37 (0.14, 0.60)**	01	**0.35 (0.13, 0.57)**	0	**0.39 (0.04, 0.74)**	01
**SO**_**2**_	**10 μg/m** ^**3**^	**0.92 (0.03, 1.81)**	6	0.90 (−1.42, 3.27)	0	−1.19 (−3.75, 1.43)	0
**O**_**3**_	**10 μg/m** ^**3**^	0.22 (−0.10, 0.54)	0	**0.16 (0.01, 0.31)**	6	0.55 (−0.41, 1.51)	07
**CO**	**1 mg/m** ^**3**^	**8.77 (2.06, 15.91)**	07	**5.38 (0.45, 10.54)**	01	**4.30 (0.14, 8.64)**	0

*Bold represents significant results. Lag0-lag7 was used to denote single-day and lag01-lag07 to denote cumulative-days.

**Figure 5:**
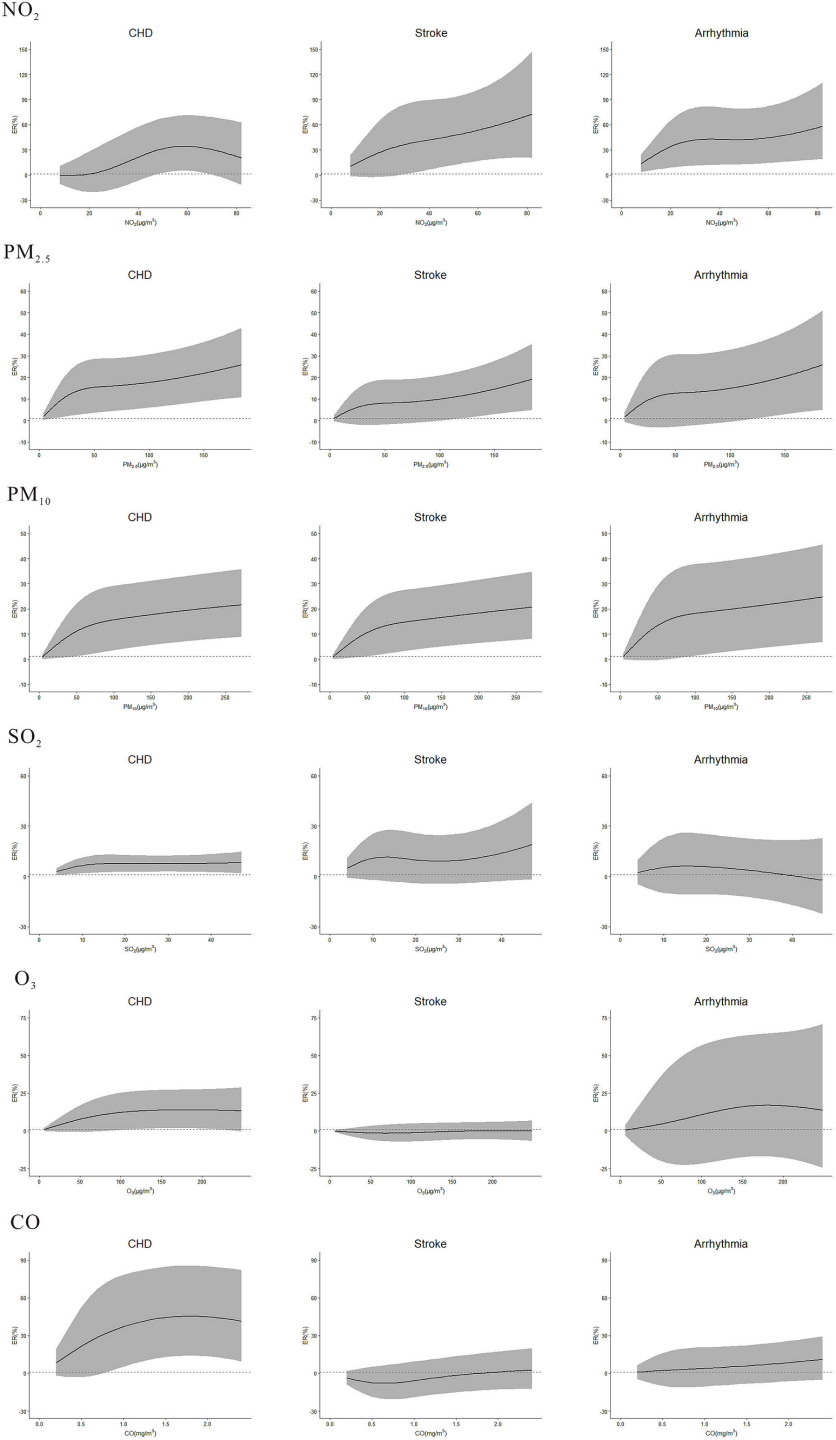
Concentration-response curve between NO_2_, PM_2.5_, PM_10_, SO_2_, O_3_, CO and the outpatient visits of CHD, stroke, and arrhythmia in single-pollutant model. The lags of NO_2_, PM_2.5_, PM_10_, SO_2_, O_3,_ and CO on outpatient visits of CHD, stroke, and arrhythmia: NO_2_-CHD (lag07), NO_2_-stroke (lag07), NO_2_-Arrhythmia (lag02), PM_2.5_-CHD (lag01), PM_2.5_-stroke (lag0), PM_2.5_-Arrhythmia (lag01), PM_10_-CHD (lag01), PM_10_-stroke (lag0), PM_10_-Arrhythmia (lag01), SO_2_-CHD (lag6), SO_2_-stroke (lag0), SO_2_-Arrhythmia (lag0), O_3_-CHD (lag0), O_3_-stroke (lag6), O_3_-Arrhythmia (lag07), CO-CHD (lag07), CO-stroke (lag01), CO-Arrhythmia (lag0).

Concentration-response curve was developed based on defining the concentration range of air pollutants from 0 to *P_99_* to mitigate the impact of extreme values. Overall, the curves had no apparent threshold. Specifically, in low-concentration range, the curves were steep, while in high-concentration range, they tended to become less steep (Figure 5). In subgroup analysis, the risks of NO_2_ on CHD, stroke, and arrhythmia, PM_2.5_ and PM_10_ on stroke, and CO on stroke and arrhythmia were stronger during cold season than warm season (Table 3). No difference was found in gender and age subgroups. Sensitivity analyses of NO_2_, PM_2.5_, PM_10_, SO_2_, O_3_ and CO on CVDs were robust after changing the *df* (Supplementary Table S2), except that the relationship between CO and stroke became weakly insignificant (ER = 4.79, 95%CI: −0.11, 9.92) after adjusting the *df* of *ns* to 8.

**Table 3:** Excess risks (95%CI) and the lags in outpatient visits of CHD, stroke, and arrhythmia associated with PM_2.5_, PM_10_, NO_2_, SO_2_, O_3_, and CO that stratified by gender, age, and season, in single-pollutant model, respectively.

Pollutant, outpatient visits	Lag (day)	Gender			Age (year)			Season		
		Male	Female	*P* value	<60	≥60	*P* value	Warm	Cold	*P* value
NO_2_										
CHD	07	4.20 (2.22, 6.22)	5.57 (3.56, 7.61)	0.347	4.98 (2.98, 7.03)	4.98 (2.87, 7.14)	1.000	−1.14 (−4.37, 2.20)	6.65 (3.88, 9.51)	**<0.001**
Stroke	07	4.68 (2.01, 7.43)	4.73 (2.20, 7.32)	0.984	4.61 (1.97, 7.32)	4.80 (2.11, 7.58)	0.920	−5.25 (−8.96, -1.40)	7.31 (3.98, 10.76)	**<0.001**
Arrhythmia	02	1.90 (−0.39, 4.24)	2.38 (0.26, 4.56)	0.984	2.59 (0.28, 4.96)	1.85 (−0.44, 4.19)	0.660	−2.90 (−6.35, 0.67)	4.38 (1.50, 7.34)	**0.002**
PM_2.5_										
CHD	01	0.85 (0.36, 1.33)	0.86 (0.37, 1.35)	0.968	0.88 (0.38, 1.37)	0.84 (0.33, 1.36)	0.928	0.52 (−0.46, 1.50)	0.79 (0.18, 1.41)	0.646
Stroke	0	0.43 (−0.70, 1.58)	−0.48 (−1.53, 0.57)	0.246	0.62 (0.08, 1.15)	0.58 (0.05, 1.12)	0.928	−0.55 (−1.47, 0.37)	0.72 (0.13, 1.31)	**0.023**
Arrhythmia	01	0.80 (0.03, 1.58)	0.87 (0.15, 1.59)	0.897	0.74 (−0.05, 1.54)	0.91 (0.15, 1.68)	0.764	0.25 (−1.32, 1.83)	0.96 (0.10, 1.82)	0.441
PM_10_										
CHD	01	0.37 (0.13, 0.61)	0.37 (0.14, 0.61)	0.968	0.44 (0.20, 0.68)	0.33 (0.08, 0.58)	0.535	0.06 (−0.35, 0.46)	0.33 (0.02, 0.64)	0.303
Stroke	0	0.51 (−0.03, 1.05)	0.22 (−0.30, 0.74)	0.447	0.37 (0.11, 0.63)	0.33 (0.08, 0.59)	0.834	−0.17 (−0.59, 0.23)	0.35 (0.06, 0.64)	**0.039**
Arrhythmia	01	0.38 (−0.01, 0.77)	0.40 (0.04, 0.76)	0.948	0.31 (−0.09, 0.70)	0.45 (0.07, 0.84)	0.603	−0.14 (−0.80, 0.53)	0.54 (0.10, 0.99)	0.095
SO_2_										
CHD	6	0.79 (−0.12, 1.71)	1.01 (0.10, 1.94)	0.741	0.76 (−0.16, 1.69)	1.02 (0.06, 2.00)	0.696	2.10 (0.78, 3.44)	0.84 (−0.34, 2.03)	0.165
Stroke	0	1.90 (−0.76, 4.63)	0.02 (−2.48, 2.57)	0.318	1.83 (−0.83, 4.55)	0.15 (−2.49, 2.86)	0.386	−2.89 (−6.37, 0.72)	1.37 (−1.78, 4.62)	0.081
Arrhythmia	0	−0.86 (−3.74, 2.11)	−1.47 (−4.13, 1.25)	0.761	3.35 (−2.59, 9.65)	−3.07 (−7.95, 2.07)	0.110	−0.50 (−4.77, 3.95)	−2.73 (−6.11, 0.77)	0.430
O_3_										
CHD	0	0.13 (−0.20, 0.46)	0.28 (−0.05, 0.62)	0.528	0.24 (−0.09, 0.58)	0.20 (−0.14, 0.55)	0.875	0.05 (−0.25, 0.36)	0.46 (−0.30, 1.22)	0.333
Stroke	6	0.16 (−0.01, 0.34)	0.16 (0.00, 0.33)	0.984	0.08 (−0.10, 0.25)	0.23 (0.05, 0.40)	0.226	0.13 (−0.02, 0.28)	0.09 (−0.27, 0.45)	0.841
Arrhythmia	07	0.37 (−0.71, 1.46)	0.69 (−0.30, 1.70)	0.669	0.29 (−0.76, 1.38)	0.76 (−0.32, 1.84)	0.552	0.37 (−0.65, 1.40)	0.59 (−1.76, 3.00)	0.868
CO										
CHD	07	6.07 (−0.19, 12.72)	7.86 (1.49, 14.64)	0.704	10.09 (3.01, 17.66)	7.92 (0.76, 15.61)	0.682	−3.09 (−14.58, 9.94)	10.55 (1.79, 20.07)	0.087
Stroke	01	5.02 (−0.57, 10.92)	5.67 (0.32, 11.31)	0.873	7.72 (1.98, 13.77)	3.61 (−1.93, 9.46)	0.327	−17.21 (−23.82, −10.03)	9.36 (2.80, 16.35)	**<0.001**
Arrhythmia	0	3.68 (−0.92, 8.50)	4.83 (0.45, 9.40)	0.726	5.40 (0.50, 10.54)	3.46 (−1.05, 8.18)	0.575	−6.27 (−13.56, 1.63)	5.24 (−0.03, 10.78)	**0.018**

*Lag0-lag7 was used to denote single-day and lag01-lag07 to denote cumulative-days.

#### 3.2.3 Double-pollutant model

Considering the strong correlation between PM_2.5_ and PM_10_, these two pollutants were not included simultaneously in double-pollutant model. NO_2_ was positively associated with CHD and stroke when controlling for other air pollutants (Table 4), and CO was also positively related with CHD (except for adjustment of NO_2_). NO_2_ and CO were associated with arrhythmia after controlling for SO_2_. The effects of PM_2.5_ and PM_10_ on CVDs remained significant after adjusting for SO_2_ and O_3_, but turned out to be insignificant after adjustment of NO_2_ and CO.

**Table 4:** Excess risks (95%CI) and the lags of NO_2_, PM_2.5_, PM_10_, SO_2_, O_3_, and CO on outpatient visits of CHD, stroke, and arrhythmia with adjustment for other pollutants in double-pollutant model, respectively.

Pollutant (adjusted)	CHD		Stroke		Arrhythmia	
	ER (95%CI)	Lag (day)	ER (95%CI)	Lag (day)	ER (95%CI)	Lag (day)
NO_2_						
PM_2.5_	**7.45 (4.93, 10.03)**	07	**8.72 (5.68, 11.84)**	07	1.61 (−0.93, 4.20)	02
PM_10_	**7.55 (5.03, 10.13)**		**7.99 (5.00, 11.06)**		1.44 (−0.96, 3.89)	
SO_2_	**8.70 (6.06, 11.41)**		**8.91 (5.78, 12.13)**		**5.58 (2.86, 8.37)**	
O_3_	**5.16 (3.17, 7.20)**		**5.04 (2.63, 7.50)**		1.99 (−0.08, 4.10)	
CO	**7.50 (4.42, 10.67)**		**6.91 (3.23, 10.72)**		2.09 (−0.81, 5.08)	
PM_2.5_						
NO_2_	0.31 (−0.26, 0.89)	01	0.19 (−0.36, 0.75)	0	0.58 (−0.26, 1.43)	01
SO_2_	**1.10 (0.56, 1.65)**		**0.68 (0.15, 1.21)**		**1.50 (0.71, 2.30)**	
O_3_	**0.84 (0.35, 1.33)**		**0.63 (0.15, 1.11)**		**0.74 (0.02, 1.46)**	
CO	0.56 (−0.17, 1.28)		0.35 (−0.33, 1.02)		0.72 (−0.33, 1.78)	
PM_10_						
NO_2_	0.06 (−0.21, 0.33)	01	0.17 (−0.08, 0.42)	0	0.27 (−0.13, 0.67)	01
SO_2_	**0.43 (0.17, 0.68)**		**0.36 (0.12, 0.60)**		**0.64 (0.26, 1.02)**	
O_3_	**0.37 (0.13, 0.60)**		**0.35 (0.13, 0.57)**		**0.36 (0.01, 0.71)**	
CO	0.19 (−0.10, 0.47)		0.24 (−0.02, 0.49)		0.30 (−0.13, 0.72)	
SO_2_						
NO_2_	−0.57 (−2.07, 0.97)	6	−2.31 (−5.30, 0.77)	0	−4.82 (−8.12, -1.40)	0
PM_2.5_	0.40 (−0.89, 1.70)		−0.84 (−3.40, 1.80)		−3.58 (−6.41, −0.65)	
PM_10_	0.50 (−0.77, 1.79)		−0.39 (−2.82, 2.11)		−2.74 (−5.47, 0.06)	
O_3_	0.06 (−1.11, 1.24)		0.72 (−1.63, 3.13)		−1.50 (−4.10, 1.18)	
CO	−0.63 (−2.02, 0.78)		−1.19 (−3.94, 1.64)		−3.88 (−6.90, −0.77)	
O_3_						
NO_2_	0.11 (−0.22, 0.43)	0	0.12 (−0.03, 0.28)	6	0.60 (−0.39, 1.59)	07
PM_2.5_	0.05 (−0.28, 0.38)		0.15 (−0.01, 0.31)		0.51 (−0.51, 1.53)	
PM_10_	0.15 (−0.17, 0.47)		**0.17 (0.02, 0.33)**		0.50 (−0.48, 1.49)	
SO_2_	0.20 (−0.13, 0.53)		**0.16 (0.00, 0.32)**		0.86 (−0.15, 1.87)	
CO	0.10 (−0.23, 0.43)		0.11 (−0.05, 0.27)		0.48 (−0.53, 1.50)	
CO						
NO_2_	−9.97 (−18.59, −0.43)	07	−2.88 (−9.32, 4.02)	01	2.66 (−3.20, 8.88)	0
PM_2.5_	**21.80 (9.52, 34.47)**		5.52 (−1.62, 13.17)		0.57 (−5.41, 6.93)	
PM_10_	**13.99 (4.53, 24.32)**		4.31 (−1.43, 10.38)		2.60 (−2.27, 7.72)	
SO_2_	**13.89 (4.96, 23.57)**		**8.43 (2.34, 14.89)**		**8.82 (3.59, 14.31)**	
O_3_	**9.14 (2.11, 16.65)**		**6.32 (1.15, 11.76)**		3.67 (−0.64, 8.16)	

*Bold represents significant results. Lag0-lag7 was used to denote single-day and lag01-lag07 to denote cumulative-days.

## 4 Discussion

NO_2_, PM_2.5_, PM_10_, SO_2_, and CO were positively correlated with each other. In December 2021, Zibo municipal government declared that traditional industries such as chemical materials and products manufacturing industry, non-metallic mineral and products industry accounted for 74% of industrial production in Zibo. Industrial furnaces and kilns in Zibo, accounting for more than one-fifth in Shandong Province, were the main sources of NO_x_. The industries of foundry, ferroalloy, cement, brick and lime could emit PMs. SO_2_ and CO were generated during the combustion of energy sources such as coal and fuel oil in industrial manufacturing. Therefore, it should be emphasized that the use of clean energy, improvement of equipment, energy conservation and emission reductions are essential.

The strongest associations between NO_2_ and CHD, stroke, arrhythmia were at lag07 (ER = 4.97, 95%CI: 3.03, 6.94), lag07 (ER = 4.71, 95%CI: 2.37, 7.10), lag02 (ER = 2.16, 95%CI: 0.12, 4.25), in single-pollutant model, respectively. The findings were consistent with previous studies, which showed significant correlations between NO_2_ and hospitalizations of CVDs (6), CHD (20) and stroke (16). Additionally, Santos reported an increase in emergency room visits of arrhythmias associated with NO_2_ (21), and Zhu identified a significant correlation between NO_2_ and cardiovascular mortality (22). A cohort study in Korea indicated that NO_2_ increased the risk of atrial fibrillation (23). In contrast, Folino’s research showed no significant association between gaseous pollutants and the occurrence of ventricular tachycardia or ventricular fibrillation (24), which differed from our study. A study indicated a significant association between exposure to NO_2_ and elevated levels of interleukin-6 (IL-6) among infants (25). Moreover, long-term exposure to NO_2_ was significantly associated with higher levels of triglycerides and high-sensitivity C-reactive protein (hs-CRP) (26). IL-6 and hs-CRP are representative circulating biomarkers of the inflammatory response in cardiovascular system. There was increasing evidence suggesting that inflammation was associated with an increased risk of CVDs (27, 28).

The highest risks of PM_2.5_ on CHD, stroke and arrhythmia were at lag01 (ER = 0.85, 95%CI: 0.38, 1.33), lag0 (ER = 0.59, 95%CI: 0.13, 1.06) and lag01 (ER = 0.84, 95%CI: 0.15, 1.53), and for PM_10_, the strongest ERs were at lag01 (ER = 0.37, 95%CI: 0.14, 0.60), lag0 (ER = 0.35, 95%CI: 0.13, 0.57) and lag01 (ER = 0.39, 95%CI: 0.04, 0.74), in single-pollutant model, respectively. There were studies that concluded significant associations between PMs and cardiovascular mortality (29), myocardial infarction death (30), incidence and daily hospital admissions of stroke (31, 32), incidence and emergency room visits of arrhythmia (21, 23). The associations between PMs and CHD, stroke, arrhythmia can be explained through the following physiological mechanisms. Firstly, PMs can initiate systemic inflammation and vascular endothelial damage, which can ultimately induce atherogenesis (33). Secondly, exposure to PMs may cause systemic inflammation and oxidative stress, promoting vasoconstriction and platelet activation (34). In addition, the health effects of PMs vary according to particle size (35), with smaller diameter particles being more likely to reach and penetrate the bronchial tubes and even cross the air-blood barrier into the circulatory system (36), thereby triggering a series of cardiovascular events. In this study, PM_2.5_ had stronger effects on CVDs than PM_10_, which was consistent with the mechanisms.

The strongest risk of SO_2_ on CHD in single-pollutant model was at lag6 (ER = 0.92, 95%CI: 0.03, 1.81). A study also revealed that exposure to SO_2_ was significantly associated with CHD (20). Moreover, studies suggested that SO_2_ increased the risk of cardiovascular emergency department visits and hospital admissions (6, 37). No association was found between SO_2_ and outpatient visits of stroke and arrhythmia. Therefore, more in-depth studies are needed to confirm the relationship between SO_2_ and CVDs.

Significant effect was observed between O_3_ and stroke in single-pollutant model, with the strongest ER at lag6 (ER = 0.16, 95%CI: 0.01, 0.31), but there was no significant association between O_3_ and CHD and arrhythmia. Exposure to lower concentration of O_3_ had no effect on mitochondrial DNA (mt-DNA) copy number. Moderate concentration of O_3_ could damage mitochondrial structure, stimulating the production of endogenous reactive oxygen species (ROS), and releasing the mt-DNA into peripheral blood. Moreover, high concentration of O_3_ might induce mitochondrial dysfunction and a reduction in mt-DNA levels (38). An animal study revealed that exposure to O_3_ caused damage of vascular endothelial and atherosclerosis in mice (39). However, a low dose of O_3_ can modulate the effective antioxidant capacity within the bloodstream and reactivate the antioxidant system (40). Only the association between O_3_ and stroke was found in this study, thus further investigations are necessary regarding the effects between O_3_ and CVDs.

CO had the highest associations with CHD, stroke and arrhythmia at lag07 (ER = 8.77, 95%CI: 2.06, 15.91), lag01 (ER = 5.38, 95%CI: 0.45, 10.54), and lag0 (ER = 4.30, 95%CI: 0.14, 8.64), in single-pollutant model. Several studies have demonstrated significant associations between CO and cardiovascular outcomes, including mortality (7, 9), hospitalization (8, 10) and outpatient visits (12). For example, a study in Yichang revealed that an increase of 1 mg/m^3^ in CO was associated with 39.30% increase in daily outpatient visits of CVDs (12). Franck’s research revealed that both CO and NO_2_ had adverse effects on cardiovascular admissions (41). An animal experiment discovered that exposure to CO in healthy rats, under experimental conditions simulating urban CO pollution, resulted in the promotion of cardiac remodeling and ventricular arrhythmia (42). CO could rapidly combine with hemoglobin to form carboxyhemoglobin, leading to tissue hypoxia (43). Exposing to CO was associated with CVDs through the following biological mechanisms, including blood coagulation (resulting in elevated fibrinogen levels) (44, 45), inflammatory response (leading to increased IL-6 levels) (46) and oxidative stress (causing an increase in ROS production) (47).

The concentration-response curve of NO_2_, PM_2.5_, PM_10_, SO_2_, O_3_ and CO on CVDs, had no apparent threshold. Specifically, in low-concentration range, the curves were steep, while in high-concentration range, they tended to become less steep. A study about hourly air pollutants and acute coronary syndrome onset in 1.29 million patients, also found that there was no obvious threshold between pollutants and acute coronary syndrome (14). It is suggested that controlling the concentration of air pollutants at low levels may achieve better effectiveness.

In subgroup analysis, the risks of NO_2_ on CHD, stroke and arrhythmia, PM_2.5_ and PM_10_ on stroke, CO on stroke and arrhythmia were stronger in cold season than warm season, which was consistent with the study of Ying (37) and Wong (48). A reasonable explanation was that the concentration of NO_2_, PM_2.5_, PM_10_ and CO was higher in cold season compared to warm season due to cold-season heating. Several studies have reported that females and the older adult were more susceptible (16, 18, 49–52), however, no difference was found in subgroup analyses of gender and age in our study, which was consistent with previous findings (8, 53). Therefore, the entire population in Zibo, especially in cold season, protective measures were needed to prevent the effect of ambient pollution. For example, reduce outdoor activities and close windows and doors in cold season when pollution is high, wear a mask when going outside, and wash hands and change clothes when returning home. In addition, further studies are needed on vulnerable populations based on age and gender. Sensitivity analyses of NO_2_, PM_2.5_, PM_10_, SO_2_, O_3_ and CO on CVDs were robust after changing the *df*, except that the relationship between CO and stroke became weakly insignificant (ER = 4,79, 95%CI: -0.11, 9.92) after adjusting the *df* of *ns* to 8. Therefore, the results of sensitivity analyses supported the associations between ambient pollutants and CVDs in the study.

In double-pollutant model, NO_2_ was positively associated with CHD and stroke. The effects of PM_2.5_ and PM_10_ on CVDs became insignificant after adjustment of NO_2_ and CO. The results were consistent with several studies. For example, a study conducted in 272 Chinese cities reported a positive association between NO_2_ and cardiovascular mortality after controlling for PM_2.5_ and CO (54). Tian suggested that the associations of NO_2_ on stroke remained significant after controlling for other air pollutants, but the effects of PM_2.5_ and CO became insignificant after adjustment of NO_2_ and SO_2_ (31). However, there was a study reported that the effect of CO on CHD mortality remained significant after adjustment of PMs, SO_2_, and NO_2_ (9). In our study, the risk of CO on CHD became insignificant after adjusting for NO_2_ and remained significant after adjusting for PMs and SO_2_. NO_2_ and CO showed much stronger effects compared with PM_2.5_, PM_10_, SO_2_, and O_3_, so NO_2_ and CO were the crucial pollutants affecting CVDs.

There were several strengths in the study. Firstly, data on 1.09 million daily outpatient visits of common CVDs in Zibo were collected over 4 years, which included cases from the entire district with extensive coverage, making the conclusions more persuasive. Secondly, specific diseases such as CHD, stroke and arrhythmia were focused on, rather than CVDs encompassing numerous diseases as a whole, making the interventions more targeted. Additionally, the outcome of this study was outpatient visits of CHD, stroke and arrhythmia, which included lots of milder patients, and therefore, outpatient visits may better reflect the acute effects of diseases, making it more sensitive compared to hospital admissions. However, the study also had limitations. Firstly, as an ecological study, ambient air pollutants were represented by daily average levels measured at monitoring stations instead of individual exposure. The study was conducted at a population level, considering that individuals had different lifestyles and physiological characteristics, which could make it difficult to accurately assess the relationships between air pollutants and CVDs. Secondly, CVD patients’ willingness and frequency of visiting hospital would decrease during the pandemic of COVID-19, which might reduce outpatient visits of CVDs, so the impact of air pollution on CVDs might be underestimated. Finally, the data on outpatient visits of CVDs were from HIS rather than community, but there was no HIS in clinics. It might underestimate the impact of air pollution on CVDs. So, the results of the research could not be extrapolated directly to other realities. Future study should be employed in community to contrast the results.

## 5 Conclusion

The study adopted single-pollutant model, concentration-response curve, subgroup analysis and double-pollutant model to investigate the association between air pollutants and outpatient visits of CVDs in Zibo. There were positive associations between NO_2_, PM_2.5_, PM_10_, CO and CVDs. While SO_2_ was only associated with CHD, and O_3_ was solely associated with stroke. NO_2_ and CO showed much stronger effects compared with PM_2.5_, PM_10_, SO_2_, and O_3_. No threshold was found between ambient pollutants and CVDs. The effects of NO_2_ on CVDs, PM_2.5_ and PM_10_ on stroke, and CO on stroke and arrhythmia were stronger in cold season than warm season.

## Data Availability

The datasets presented in this article are not readily available because data on daily outpatient visits of cardiovascular diseases were not publicly available. Requests to access the datasets should be directed to chenliang@qiluhospital.com.
