# Supplementary material for: Association between ambient air pollution and outpatient visits of cardiovascular diseases in Zibo, China: a time series analysis
Source: Front Public Health. 2025 Jan 8;12:1492056. doi: 10.3389/fpubh.2024.1492056 (PMC11750768; doi:10.3389/fpubh.2024.1492056)
Supplement: Supplementary file 1 [file Data_Sheet_1.docx]

Supplementary Material

**Table S1** Spearman’s correlation coefficients among ambient air pollutants and meteorological factors in Zibo, 2019-2022.

| **Variable** | **NO_2_** | **PM_2.5_** | **PM_10_** | **SO_2_** | **O_3_** | **CO** | **Temperature** | **Relative humidity** |
| --- | --- | --- | --- | --- | --- | --- | --- | --- |
| **NO_2_** | 1.00 |  |  |  |  |  |  |  |
| **PM_2.5_** | 0.67* | 1.00 |  |  |  |  |  |  |
| **PM_10_** | 0.70* | 0.88* | 1.00 |  |  |  |  |  |
| **SO_2_** | 0.70* | 0.61* | 0.70* | 1.00 |  |  |  |  |
| **O_3_** | -0.36* | -0.18* | -0.14* | -0.22* | 1.00 |  |  |  |
| **CO** | 0.80* | 0.82* | 0.74* | 0.70* | -0.26* | 1.00 |  |  |
| **Temperature** | -0.51* | -0.41* | -0.40* | -0.51* | 0.78* | -0.45* | 1.00 |  |
| **Relative humidity** | 0.00 | 0.02 | -0.24* | -0.36* | -0.14* | 0.07* | 0.19* | 1.00 |
| **Wind speed** | -0.47* | -0.23* | -0.10* | -0.20* | 0.17* | -0.41* | 0.13* | -0.42* |

**P*＜0.05.

**Table S2** Excess risks (95%CI) and the lags in outpatient visits of CHD, stroke, and arrhythmia after modifying the degree of freedom in single-pollutant model.

| **Pollutant** | **CHD** | |  | **Stroke** | |  | **Arrhythmia** | |
| --- | --- | --- | --- | --- | --- | --- | --- | --- |
|  | **ER (95%CI)** | **Lag (day)** |  | **ER (95%CI)** | **Lag (day)** |  | **ER (95%CI)** | **Lag (day)** |
| **NO_2_** |  |  |  |  |  |  |  |  |
| Temp/rh/wind (*df*=4) ^a^ | **5.47(3.49,7.49)** | 07 |  | **5.09(2.71,7.52)** | 07 |  | **2.73(0.67,4.83)** | 02 |
| Temp/rh/wind (*df*=5) ^b^ | **5.86(3.85,7.90)** |  |  | **5.10(2.69,7.56)** |  |  | **2.62(0.54,4.74)** |  |
| Time (*df*=8) ^c^ | **4.16(2.20,6.15)** |  |  | **3.80(1.39,6.26)** |  |  | **2.58(0.50,4.70)** |  |
| **PM_2.5_** |  |  |  |  |  |  |  |  |
| Temp/rh/wind (*df*=4) | **0.86(0.39,1.34)** | 01 |  | **0.63(0.16,1.10)** | 0 |  | **0.84(0.15,1.53)** | 01 |
| Temp/rh/wind (*df*=5) | **0.88(0.40,1.35)** |  |  | **0.62(0.15,1.09)** |  |  | **0.77(0.07,1.46)** |  |
| Time (*df*=8) | **0.78(0.32,1.24)** |  |  | **0.52(0.05,0.98)** |  |  | **0.84(0.15,1.53)** |  |
| **PM_10_** |  |  |  |  |  |  |  |  |
| Temp/rh/wind (*df*=4) | **0.39(0.16,0.62)** | 01 |  | **0.36(0.14,0.59)** | 0 |  | **0.42(0.08,0.77)** | 01 |
| Temp/rh/wind (*df*=5) | **0.41(0.18,0.64)** |  |  | **0.36(0.13,0.58)** |  |  | **0.39(0.04,0.74)** |  |
| Time (*df*=8) | **0.33(0.11,0.55)** |  |  | **0.31(0.09,0.53)** |  |  | **0.40(0.06,0.74)** |  |
| **SO_2_** |  |  |  |  |  |  |  |  |
| Temp/rh/wind (*df*=4) | **0.99(0.09,1.89)** | 6 |  | 0.95(-1.38,3.33) | 0 |  | -1.37(-3.92,1.24) | 0 |
| Temp/rh/wind (*df*=5) | **1.05(0.15,1.95)** |  |  | 0.92(-1.42,3.31) |  |  | -1.50(-4.06,1.13) |  |
| Time (*df*=8) | **0.90(0.03,1.78)** |  |  | 0.59(-1.70,2.92) |  |  | -1.22(-3.76,1.38) |  |
| **O_3_** |  |  |  |  |  |  |  |  |
| Temp/rh/wind (*df*=4) | 0.20(-0.12,0.52) | 0 |  | **0.16(0.01,0.31)** | 6 |  | 0.38(-0.58,1.34) | 07 |
| Temp/rh/wind (*df*=5) | 0.23(-0.09,0.56) |  |  | **0.16(0.01,0.31)** |  |  | 0.32(-0.64,1.29) |  |
| Time (*df*=8) | 0.25(-0.06,0.56) |  |  | **0.18(0.03,0.34)** |  |  | 0.80(-0.20,1.81) |  |
| **CO** |  |  |  |  |  |  |  |  |
| Temp/rh/wind (*df*=4) | **10.33(3.47,17.65)** | 07 |  | **5.95(0.97,11.17)** | 01 |  | **4.53(0.36,8.86)** | 0 |
| Temp/rh/wind (*df*=5) | **10.70(3.78,18.08)** |  |  | **6.02(1.02,11.26)** |  |  | **4.35(0.18,8.70)** |  |
| Time (*df*=8) | **7.32(0.74,14.32)** |  |  | 4.79(-0.11,9.92) |  |  | **4.69(0.53,9.02)** |  |

^a b^ Temp/rh/wind (*df*=4) and temp/rh/wind (*df*=5) presented the *df* for the cross-basis of temperature, relative humidity, and wind speed were conducted to 4 and 5, respectively. The *df* for the natural cubic spline (*ns*) used to control the long-term trends and seasonal trends was 7. ^c^ Time (*df*=8) presented the *df* for the cross-basis of temperature, relative humidity, and wind were conducted to 3, and the *df* for the *ns* used to control for long-term trends and seasonal trends was modified to 8. Bold represents significant results. Lag0-lag7 was used to denote single-day and lag01-lag07 to denote cumulative-days.
